# The evolution of eusociality: no risk‐return tradeoff but the ecology matters

**DOI:** 10.1111/ele.13452

**Published:** 2019-12-29

**Authors:** Jeremy Field, Hiroshi Toyoizumi

**Affiliations:** ^1^ Centre for Ecology and Conservation University of Exeter Penryn Campus Cornwall TR10 9EZ UK; ^2^ Graduate School of Accounting and Department of Applied Mathematics Waseda University Nishi‐waseda 1‐6‐1 Shinjuku Tokyo 169‐8050 Japan

**Keywords:** Bees, bet‐hedging, eusociality, Hamilton's Rule, Hymenoptera, inheritance, social behaviour, social evolution, wasps

## Abstract

The origin of eusociality in the Hymenoptera is a question of major interest. Theory has tended to focus on genetic relatedness, but ecology can be just as important a determinant of whether eusociality evolves. Using the model of Fu *et al*. (2015), we show how ecological assumptions critically affect the conclusions drawn. Fu *et al*. inferred that eusociality rarely evolves because it faces a fundamental ‘risk‐return tradeoff’. The intuitive logic was that worker production represents an opportunity cost because it delays realising a reproductive payoff. However, making empirically justified assumptions that (1) workers take over egg‐laying following queen death and (2) productivity increases gradually with each additional worker, we find that the risk‐return tradeoff disappears. We then survey Hymenoptera with more specialised morphological castes, and show how the interaction between two common features of eusociality – saturating birth rates and group size‐dependent helping decisions – can determine whether eusociality outperforms other strategies.

## Graphical abstract

Theories concerning the origin of eusociality in Hymenoptera have tended to focus on genetic relatedness, but ecology can be just as important. With realistic ecological assumptions, we show that eusociality does not face a previously postulated ‘risk‐return tradeoff’. We find that the interaction between two common features of eusociality – birth rates that saturate at large group sizes, and group‐size dependent helping decisions – can instead determine whether eusociality outperforms other strategies.

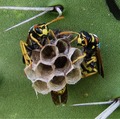

## Introduction

The origin and maintenance of eusociality is a question of major interest in evolutionary biology. In eusocial and cooperatively‐breeding taxa, some individuals, known as helpers or workers, at least temporarily forfeit their own reproduction to aid the reproduction of other individuals known as queens or breeders. The main approach used to understand the seemingly paradoxical behaviour of helpers has been inclusive fitness theory as embodied by Hamilton's Rule (Hamilton 1964). Hamilton's Rule states that an individual should help to rear the queen's offspring if *rb* > *c*, where *r* is queen‐worker genetic relatedness, *b* is the reproductive benefit to the queen of the help she receives and *c* is the cost of helping to the worker (offspring forfeited).

The eusocial Hymenoptera (ants, bees and wasps) range from ‘primitively eusocial’, where there are no fixed differences between queens and workers and individuals can switch roles, to ‘advanced eusocial’ where there may be extreme queen‐worker dimorphism and irreversibly sterile workers. Starting with Hamilton's (1964) famous three‐quarters relatedness hypothesis, theoretical attempts to understand why eusociality evolved in Hymenoptera have tended to focus on factors that can raise genetic relatedness such as haplodiploidy and more recently, lifetime queen monogamy (e.g. Trivers & Hare 1976; Seger 1983; Boomsma 2009; Fromhage & Kokko 2011; Nonacs 2011, 2014; Gardner *et al*. 2012; Quinones & Pen 2017; Rautiala *et al*. 2019). It remains equivocal, however, whether relatedness really was unusually high in the ancestors of today's eusocial taxa (Nonacs 2011; Pernu & Helantera 2019), and the ecological parameters in Hamilton's Rule are potentially just as important determinants of whether helping or solitary nesting is the optimal strategy (Queller 1994, 1996; Field *et al*. 2000; Korb & Heinze 2008; Avila & Fromhage 2015). Recent models suggest that two features of life history and ecology have a critical impact on whether eusociality evolves: the potential for workers to take over egg‐laying following the queen's death, and the relationship between group size and productivity (Fromhage & Kokko 2011; Nonacs 2011, 2019; Fu *et al*. 2015). Focussing on a recent paper by Fu *et al*. (2015), we first show how making unrealistic assumptions about these two features can produce apparently far‐reaching but incorrect conclusions. We then incorporate more realistic assumptions.

Fu *et al*. (2015) used a structured population model to compare solitary (non‐social) and eusocial genotypes in terms of basic reproductive numbers and long‐term probabilities of lineage extinction. They used Markov chain and Branching processes to model group and population dynamics respectively in continuous time with overlapping generations (cf discrete time models such as Wild 2011; Lehmann *et al*. 2016; Mullon *et al*. 2016). Fu *et al*. came to the potentially important conclusion that eusociality faces an inherent ‘risk‐return tradeoff’. By definition, eusociality entails the production of non‐reproductive worker offspring. The rationale underlying the risk‐return tradeoff is that worker production represents an opportunity cost for a eusocial queen because it delays her fitness payoff (Fu *et al*. 2015): her group may fail before reproductive offspring are produced. This cost is avoided by solitary individuals that produce entirely reproductives. The opportunity cost means that for eusociality to outperform the solitary strategy in Fu *et al*.'s model, the rewards (reproductive offspring) eventually associated with a successful eusocial group must be so large as to be unlikely. For example, in a numerical example they focus on (their fig. 2), eusociality has a higher extinction probability than the solitary strategy in about half of the parameter space, even when groups of three individuals are 20 times as productive as solitary individuals. Fu *et al*. link their findings to the apparent rarity with which eusociality has evolved in nature, and the fact that it has been lost repeatedly in some lineages of bees (Wcislo & Danforth 1997; Rehan *et al*. 2014). The existence of a fundamental risk‐return tradeoff characterising eusociality is thus an important finding worth evaluating.

We hypothesised that the ‘riskiness’ of the eusocial strategy in Fu *et al*.'s model might reflect two ecological assumptions that seem unrealistic in the early stages of eusocial evolution: (1) queen replacement is not allowed, so that a queen's death results in failure of the whole group; (2) there is a threshold group size below which workers add nothing to group productivity, and although productivity does increase at the threshold, it increases no further if group size exceeds the threshold. To more closely match hymenopteran biology, we modify these assumptions as well as a third assumption that (3) individual workers are immortal. We find that the risk‐return tradeoff then disappears. Drawing on empirical data, we next extend the model and show how two common features of more specialised eusocial groups – saturating birth rates and group size‐dependent helping decisions – interact to determine whether eusociality outperforms other strategies. We conclude that Fu *et al*.'s modelling framework is potentially useful, but the precise assumptions made about ecology are critical.

## Results

**Figure 1:**
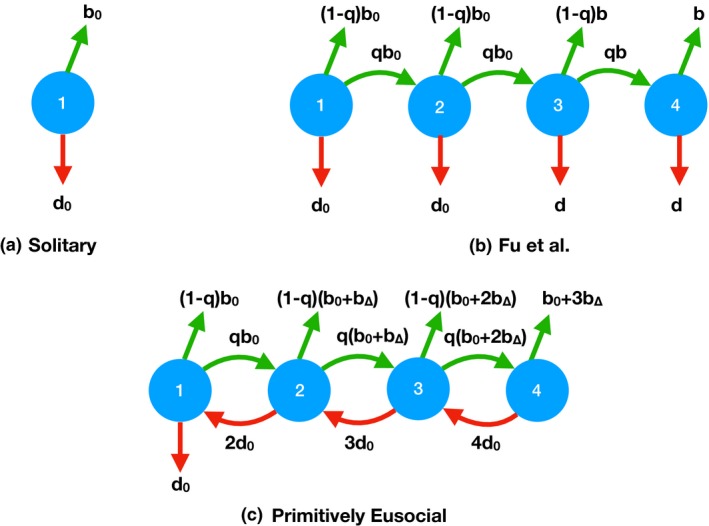
Schematic showing Markov chain dynamics assumed for (a) Solitary strategy; (b) Fu *et al*.'s eusocial strategy with a threshold group size *m* = 3; (c) our Primitively Eusocial strategy with inheritance of the queen position, birth rates that increase linearly with group size, and worker mortality. Numbers in blue circles represent group size (including the queen) from *i* = 1 to a maximum size *M* (here *M* = 4). Green arrows represent births of offspring that stay and help (at rate *q* × birth rate) or disperse (at rate (1 − *q*) × birth rate). Red arrows represent deaths of individuals (rate *d* or *d*_0_) or (downward arrows) catastrophic group failure (rate *d*_0_ or, above the threshold group size in part (b), rate *d*).

**Figure 2:**
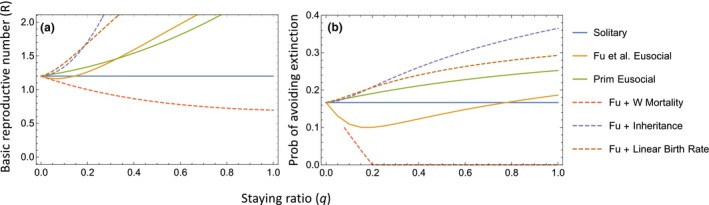
Relationship between the probability that offspring become workers (staying ratio *q*) and (a) basic reproductive numbers (R) and (b) the probability of avoiding extinction (‘emergence probability’ of Fu et al. 2015) for the models discussed in the main text: Fu *et al*.'s Solitary and Eusocial strategies; Fu *et al*.'s Eusocial strategy with each of the three assumptions discussed in the text relaxed separately (dashed lines); and our Primitively Eusocial strategy with all three assumptions relaxed simultaneously. Parameter values are similar to Fu *et al*.'s figure 2, with *M* = *m* = 3, *d*_0_ = 0.1, *d* = 0.05, *b*_0_ = 0.12, *b* = 0.45, *b_∆_* = (*b* − *b*_0_)/(*m* − 1) = 0.165.

Fu *et al*.'s basic model assumes asexuality and relatedness of 1.0, allowing them to focus on ecological effects. Group dynamics are specified as follows. Females of the solitary genotype have death rate *d*_0_ and produce offspring at rate *b*_0_. All of their offspring are reproductives (new queens) that disperse and initiate new nests alone (Fig. 1a). Reproductive females of the eusocial genotype also initiate nests alone, but in contrast with solitary females, they produce offspring that with probability *q* (the ‘staying ratio’) become life‐long workers on their natal nests (Fig. 1b). The remaining proportion 1 − *q* of offspring are dispersing reproductives. Once a maximum group size (*M*) is reached, new offspring must all disperse. By staying on their natal nests, workers can boost the reproductive success of their mother queen, both by helping to rear more offspring and by reducing the queen's death rate. Fu *et al*. model this as a threshold effect: at group size *m*, the offspring birth rate increases from *b*_0_ to *b* and the queen's death rate decreases from *d*_0_ to *d*. Death of the queen terminates the entire group, but worker mortality does not otherwise occur. Across most of the parameter space investigated, Fu *et al*. found that eusocial genotypes performed worse than solitary genotypes in terms of the chance of lineage extinction, which occurs when the initial eusocial group and all descendant groups have terminated. In addition, eusocial genotypes often had smaller basic reproductive numbers (R), where R is the number of dispersing reproductive offspring that one solitary individual or eusocial group produces in its lifetime (Fig. 2: Solitary vs. Fu *et al*. Eusocial).

We proceeded initially by modifying assumptions (1)–(3) one by one. We illustrate our findings in detail with the parameter values that Fu *et al*. focussed on, except that we use *M* = 3 because a small group size seems appropriate when modelling the origin of eusociality (Fu *et al*. focussed on *M* = 2 and especially *M* = 100). However, we obtained qualitatively similar results with larger group sizes (e.g. *M* = 10; full details of our mathematical methods are in the Supporting Information). To be consistent with Fu *et al*., we compare models in terms of both R and the extinction probability. However, we believe that R is a less reliable performance measure when modelling overlapping generations in continuous time. Particularly when queen replacement is allowed, eusocial groups tend to have far greater lifespans then solitary individuals, so that R is measured over a longer time period for eusociality, and tends to be positively correlated with group size (e.g. see Fig. 5).

### Assumption (1): queen replacement

Fu *et al*. assumed that the death of a eusocial queen results in catastrophic group failure. This assumption seems likely to contribute to the risk inherent in eusociality: when the queen dies, the lifespans of any of her offspring that chose to help are truncated, whereas offspring of solitary individuals always continue to reproduce until they die. Furthermore, queen death will tend to prevent groups from reaching the threshold group size *m* at which worker benefits kick in. The empirical examples that Fu *et al*. refer to are primitively eusocial Hymenoptera, which exhibit minimal morphological differentiation between queens and workers. This is appropriate when considering the origin and spread of a eusocial strategy, since marked morphological differentiation is unlikely to be present initially. In such taxa, workers are not irreversibly committed to their roles. Queen death does not normally lead to group failure because a worker can take over the queen position. Queen replacement is indeed the norm in the major lineages of primitively eusocial Hymenoptera where it has been studied (Nonacs 2014), including sweat bees (e.g. Yanega 1989; Michener 1990; Mueller 1991; Field *et al*. 2010), carpenter bees (e.g. Stark 1992), hover wasps (Bridge & Field 2007; Field 2008) and paper wasps (e.g. Strassmann *et al*. 2004; Tsuji & Tsuji 2005). We modified Fu *et al*.'s model to allow for queen replacement. If the current queen died in a group of size *i*, a worker was assumed to take over as queen, allowing the group to continue reproducing with the birth rate and queen death rate appropriate to a group of size *i* − 1. Group failure then occurs only if successive queens die faster than new workers are recruited. In contrast with Fu *et al*.'s findings, eusociality then has a higher basic reproductive number and lower risk of extinction than the solitary strategy (Fig. 2, Fu + Inheritance vs. Solitary).

### Assumption (2): the relationship between productivity and group size

**Figure 3:**
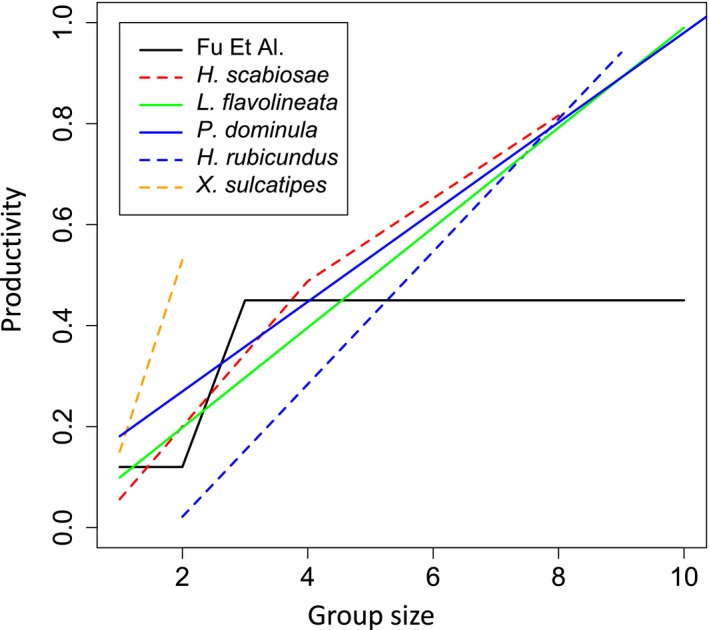
Observed relationships between group size and measures of group productivity in five species of primitively eusocial Hymenoptera, scaled so that they fit into the same plot, compared with the threshold relationship assumed by Fu *et al*. with *m* = 3 and *b*_0_ = 0.12, *b* = 0.45. Species (productivity measure and scaling) as follows: sweat bee *Halictus scabiosae* (number of second brood offspring/5; Brand & Chapuisat 2014); hover wasp *Liostenogaster flavolineata* (daily offspring hatch rate × 9; Shreeves & Field 2002); paper wasp *Polistes dominula* (number of brood in cofoundress nests/50; Shreeves et al. 2003); sweat bee *Halictus rubicundus* (number of second brood offspring/15; Yanega 1989); carpenter bee *Xylocopa sulcatipes* (number of offspring/10 in 1986; Stark 1992)

The second of Fu *et al*.'s assumptions that we modify has two parts. The first part is that a eusocial queen's reproductive rate remains the same as a solitary female's reproductive rate (*b*_0_) until a threshold group size *m* is reached. Only at group size *m* does the birth rate increase to *b*. The second part is that if group size exceeds *m* as more workers are recruited, the birth rate does not increase beyond *b*. Using a threshold *m* again seems likely to contribute to the risk associated with eusociality: workers add nothing to the queen's productivity if the group fails before the threshold group size is reached, and additional workers above the threshold contribute nothing unless group size falls back down to *m*. In contrast, the offspring of solitary females are assumed to reproduce continuously from birth. In reality, in all of the major lineages of primitively eusocial wasps and bees, the queen's reproductive rate normally increases gradually, often linearly, with each additional worker (e.g. Yanega 1989; Shreeves & Field 2002; Brand & Chapuisat 2014). And the queen's reproductive rate increases in the presence of just one or two workers, rather than only above a threshold group size (Fig. 3). This reflects worker foraging to provision the queen's offspring at all group sizes, and the fact that the nest is guarded almost continuously once at least one worker is present. These are both advantages likely to have operated even at the evolutionary origin of eusociality (e.g. Queller 1994). If we allow productivity to increase by the same amount (*b_∆_*) for each additional worker irrespective of group size, both the basic reproductive number and the probability of avoiding extinction become higher for eusociality than for solitary nesting, even without allowing inheritance (Fig. 2, Fu + Linear Birth Rate vs. Solitary). By immediately boosting the reproductive success of the queen at all group sizes, each worker is now reproducing as efficiently as the dispersing offspring of a solitary female, or more efficiently if per capita productivity increases with increasing group size (as in our Fig. 2 where *b_∆_* > *b*_0_). Another possibility, which would at least partially remove the disadvantage that a threshold group size causes for eusociality, would be if offspring stay and help in the first place only when their groups are small (Appendix IIA in Fu *et al*. 2015, Liao *et al*. 2015). There is some evidence for this, especially in large‐colony taxa (see below), but the only experimental test in a primitively eusocial hymenopteran provided no evidence that staying decisions were group size‐dependent (Field *et al*. 1999).

### Assumption (3): worker mortality

The third assumption we modified is that workers are immortal except when catastrophic whole‐nest failure occurs. We added worker mortality to Fu *et al*.'s model, so that individual workers had the same death rate (*d*_0_) as solitary females (e.g. Field *et al*. 2000). As expected given that only eusocial nests have workers, allowing worker mortality (while not allowing inheritance and retaining a threshold group size before productivity increases) is disadvantageous for eusociality compared with solitary nesting (Fig. 2: Fu + W mortality).

### Combining all three modifications of the model

**Figure 4:**
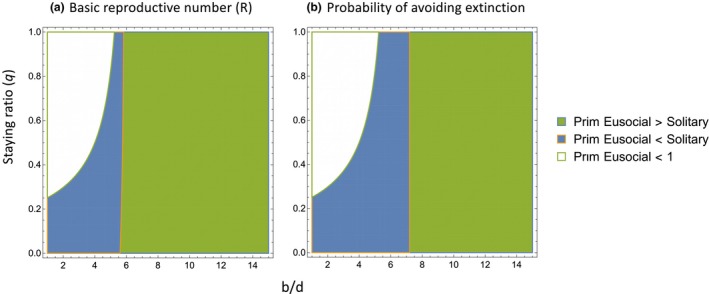
Performance of our Primitively Eusocial strategy compared with the solitary strategy in relation to the staying ratio (*q*) and the birth/death ratio (*b*/*d*). Performance is measured in terms of (a) basic reproductive number (R) and (b) the probability of avoiding extinction. Parameter values are *M* = *m* = 3, *d*_0_ = 0.1, *d* = *d*_0_ *i*, *b*_0_ = 0.12, *b_∆_* = (*b* − *b*_0_)/(*m* − 1). In (b), there is a threshold *b*/*d* value (7.2, vertical orange line) at which a worker increases the queen's productivity by an amount equal to the productivity of a solitary female (i.e. *b_∆_* = *b*_0_ = 0.12, which requires *b* = 0.36 so that *b*/*d* = 7.2). Above this threshold (green area), workers are more productive than solitary females and the Primitive Eusocial strategy performs best. Below the threshold (blue + white areas), the solitary strategy performs best. In the white area, the Primitively Eusocial strategy has R < 1 (guaranteeing extinction) in (a), or has a zero chance of avoiding extinction in (b).

Allowing queen replacement in Fu *et al*.'s model, or switching from a threshold to a linear increase in the birth rate with increasing group size, results in eusociality outperforming solitary nesting when the maximum group size is 3 (Fig. 2) or 10 (Supporting Information). Adding just worker mortality has the opposite effect. We lastly combined all three of these modifications to produce an overall, ecologically more realistic Primitively Eusocial model (Fig. 1c). With this model and our illustrative parameter values, the risk‐return tradeoff that Fu *et al*. highlighted disappears: at all staying ratios, eusociality has higher basic reproductive numbers and lower extinction probabilities than solitary nesting (Fig. 2, Primitively Eusocial vs. Solitary). The larger the staying ratio (*q*), the larger the advantage that eusociality has (assuming *b_∆_* > *b*_0_; Fig. 2). This contrasts with Fu *et al*.'s finding that eusociality always performs worse than solitary nesting, or (depending on parameter values) performs better only at intermediate staying ratios (e.g. Fig. 2, Fu *et al* Eusocial vs. Solitary) or when workers have a huge positive impact on the offspring birth rate. Beyond the precise parameter values used in Fig. 2, Fig. 4 generalises our findings to the larger areas of parameter space investigated by Fu *et al*. (see also Fig. S5 in Supporting Information).

### The origin of more specialised eusocial taxa

Even if they are not applicable to the origin of eusociality, could the ecological assumptions underlying the risk‐return tradeoff apply to the origin of more specialised castes in taxa such as honeybees and ants? Such taxa usually have significant queen‐worker dimorphism so that workers cannot become fully functional queens (Hölldobler & Wilson 1990; Bourke & Franks 1995; Peeters & Molet 2010). Below, we survey the empirical literature in relation to Fu *et al*.'s assumptions, and show how two common features of the relationship between group size and productivity in more specialised taxa can interact to determine extinction probabilities.

#### Queen replacement and consequences of queen death in more specialised taxa

Even though a worker cannot become a fully functional replacement queen in taxa with morphologically specialised castes, queen death typically does not completely truncate the productive lives of remaining workers as assumed in Fu *et al*.'s models. In some such taxa, complete queen loss is anyway unlikely because there are multiple queens per group, and because additional daughter queens can be adopted if necessary (Bourke & Franks 1995; Keller 1995; Peeters & Molet 2010). Even in species with only a single queen, however, there are mechanisms that allow workers remaining after queen death to be productive. First, workers can often rear a replacement queen (e.g. honeybees, stingless bees and some ants; Bourke & Franks 1995; Hatch *et al*. 1999; Miller & Ratnieks 2001; Faustino *et al*. 2002; Clemencet *et al*. 2008; Alves *et al*. 2009), or daughter queens already present can be adopted as replacements (e.g. Tschinkel & Howard 1978; Tschinkel 1996; Peeters & Molet 2010). Second, although specialised hymenopteran workers are often unable to mate and lay female eggs, they can usually still lay unfertilised male eggs. Thus, even if queen death is followed by gradual die‐off of existing workers and eventual group extinction, remaining workers can produce their own male reproductives (e.g. bumblebees, honeybees, vespine wasps, many ants: Miller & Ratnieks 2001; Dijkstra & Boomsma 2007; Smith *et al*. 2007; Peeters & Molet 2010; Almond *et al*. 2019) as well as rear remaining offspring of the queen through to adulthood (many ants: Dijkstra & Boomsma 2006, 2007).

The closest approximation to Fu *et al*.'s assumption about queen death may be the minority of taxa (*c*. 9 ant genera and *Frieseomelitta* stingless bees) where there is a single queen and completely sterile workers that cannot lay eggs of either sex. Some of the above mechanisms may still operate, however (e.g. adoption of a daughter queen; Tschinkel & Howard 1978; Tschinkel 1996), along with additional taxon‐specific mechanisms such as fusion with a related colony after queen death (Kronauer *et al*. 2010; see also Peeters & Molet 2010).

The mechanisms above mean that queen death will not usually lead to catastrophic group failure in Hymenoptera with morphologically specialized workers. Furthermore, even if there was partial truncation of worker productivity, for example if offspring production is delayed while a new queen is reared (e.g. Tschinkel & Howard 1978; Miller & Ratnieks 2001; Faustino *et al*. 2002), its effect on the probability of extinction would depend on queen mortality rates (Section 8 and Fig. S11 of Supporting Information). In advanced eusocial taxa with only a single queen, queens tend to be extremely long‐lived, reflecting the protected environment within their nests (e.g. 20 year queen lifespan: Pamilo 1991; see also Keller & Genoud 1997; Peeters & Molet 2010). In ecological situations where individual queens have higher mortality rates, so that any truncation of worker productivity might otherwise be more frequent, there tend to be multiple queens per group (Keller & Genoud 1997).

#### The relationship between group size and productivity in more specialised taxa

**Figure 5:**
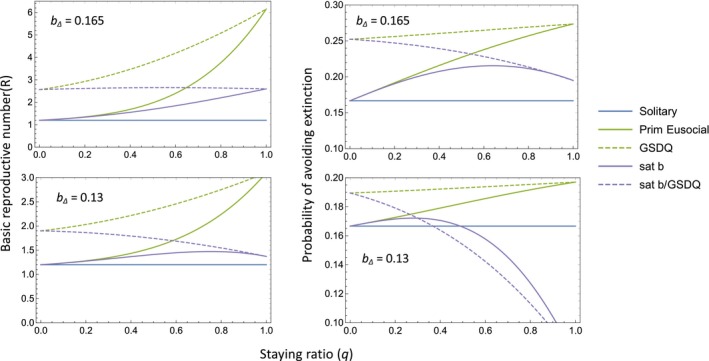
Comparison of basic reproductive numbers, R, and the probability of avoiding extinction (‘emergence probability’ of Fu et al. 2015) for solitary vs. eusocial strategies when *b_∆_* = 0.165 (upper graphs) and *b_∆_* = 0.13 (lower graphs). Models shown are the Primitively Eusocial model presented earlier (solid green line) and the same model with the following modifications: a birth rate that saturates at group size *m* (solid purple line; *b* = *b*_0_ + *b_∆_* (*m* − 1) for *i* ≥ *m*); a GSDQ strategy where q = 1 when group size *i* < *m* and q takes the value on the *x*‐axis when *m* ≤ *i* < *M* (dashed green line); both a saturating birth rate and a GSDQ strategy (dashed purple line). For all models, q = 0 when *i* = *M*. Other parameter values: *m* = 3, *M* = 5, *d*_0_ = 0.1, *d* = *d*_0_ *i*, *b*_0_ = 0.12. Note that when q = 0, all strategies except those involving GSDQ are effectively solitary. Basic reproductive numbers are noticeably less informative than extinction rates here. For example, R is positively correlated with q under a saturating birth rate, because groups persist for longer when more offspring choose to become workers, even though the probability of lineage extinction is actually increasing.

In advanced eusocial taxa, there is also little evidence for Fu *et al*.'s second assumption, that groups must reach a certain size before workers can boost the queen's productivity. However, the relationship between group size and productivity often differs from that assumed for our Primitively Eusocial strategy (Fig. 4). Although productivity (including both worker and reproductive offspring) increases linearly with group size in some advanced eusocial species (as assumed in Fig. 4), it is probably commoner for the relationship to saturate in larger groups (Michener 1964; Tschinkel 1993; Wagner & Gordon 1999; Kramer *et al*. 2014). Saturating (declining) birth rates might occur if larger groups exhaust local resources, are more attractive to natural enemies, or waste more resources in within‐group conflict, so that productivity per individual eventually declines (Poitrineau *et al*. 2009; Kramer *et al*. 2014). Fu *et al*.'s (2015) models included an extreme version of birth rate saturation, where the birth rate stayed constant above group size *m*. Not surprisingly, this increases the extinction probability (Fig. 5). Especially at high staying ratios (q), where groups more often reach the size where additional workers have no effect on productivity, the chance of avoiding extinction may then be smaller than for our Primitively Eusocial strategy (e.g. Fig. 5 with *b_∆_* = 0.13, ‘sat b’ line). However, another widespread feature of social insect group dynamics, a group size‐dependent staying ratio (GSDQ), has the potential to mitigate this effect. GSDQ means that groups initially produce mainly or entirely worker offspring (high q), and switch to producing reproductives (lower q) only once they reach a certain size. This is typical of more specialized eusocial taxa such as honeybees, yellowjacket wasps (Vespinae) and ants, as well some primitively eusocial lineages such as sweat bees and paper wasps (e.g. Tschinkel 1993; Bourke & Franks 1995; Strohm & Bordon‐Hauser 2003; Peeters & Molet 2010; Leadbeater *et al*. 2011). Fu *et al*.'s (2015) basic asexual model included a component of GSDQ in that all offspring were assumed to become new reproductives (q = 0) once a maximum group size (*M*) was reached. However, this group size was larger than the group size (*m*) at which the birth rate saturated, leading to the production of workers that added nothing to group productivity (at group sizes *m* < *i* < *M*). We can instead model a situation where staying decisions are linked to an offspring's expected productivity as a worker: all offspring become workers while productivity is still increasing linearly below the saturation point (q_i<sat_ = 1), but a proportion become reproductives once group size reaches the saturation point (q_i≥sat_ < 1). The extinction probability can then actually be lower than for the solitary strategy or the simple Primitively Eusocial strategy we presented earlier (Fig. 5, sat b/GSDQ line). This tends to be the case when the GSDQ switch is more extreme, that is, when almost all offspring become reproductives above the saturation point (q_i≥sat_ is low, as well as *b_∆_* > *b*_0_; see also fig. S1 of Fu *et al*. 2015 assuming threshold functions). It is notable that if the birth rate does not saturate, but remains linear even at larger group sizes (*b_∆_* > *b*_0_ and *b_∆_* remains constant), as in many primitively eusocial (Fig. 3) and probably some advanced eusocial taxa (e.g. Kramer *et al*. 2014), the GSDQ strategy outperforms our simple Primitively Eusocial strategy at all values of *q* (Fig. 5, GSDQ line).

## Discussion

### The origin of eusociality

In our Primitively Eusocial model, just as in the models of Fu *et al*. (2015), a proportion of offspring become workers instead of being the reproductives that solitary females produce. However, so long as the positive effect that each worker has on the queen's productivity at least matches the productivity of a dispersing offspring produced by a solitary female, the basic reproductive numbers and extinction probabilities for eusociality are at least as favourable as for solitary nesting (Fig. 4). These conditions are broadly similar to those predicted to favour helping using Hamilton's Rule, with r = 1 in these asexual models. Our analyses suggest that the disadvantage inferred for eusociality by Fu *et al*. stemmed from unrealistic assumptions that reduced or delayed the contribution that workers make to group productivity. In fact, there are reasons to think that it is dispersing offspring of solitary genotypes, rather than worker offspring of eusocial genotypes, that will experience the greater delay in realising their productivity in nature. Unlike workers, dispersing offspring must find a nest site and initiate a nest before they can begin reproducing. Dispersing offspring must start rearing the youngest possible brood (eggs) in a new nest, whereas workers can often begin rearing older offspring that are already present in their natal nests (reproductive head‐start: Queller 1989, see also Avila & Fromhage 2015). Furthermore, unlike a solitary female, the investment that a worker makes in rearing offspring may not be completely wasted if she dies young: other group members may be able to bring the part‐reared offspring that she contributed to through to adulthood (Gadagkar 1990; Field *et al*. 2000). In contrast, the death of a solitary female usually leads to the total failure of any part‐reared brood (Field *et al*. 2000; Shreeves *et al*. 2003).

Are there empirical exceptions to our evaluation of the assumptions made by Fu *et al*.? Although group productivity increases gradually with group size in primitively eusocial Hymenoptera, the increase may not always be linear. For example, Brand & Chapuisat (2014) found that the first 4 workers each added a similar amount to the queen's productivity in the sweat bee *Halictus scabiosae* (Rossi), while additional workers beyond 4 had a smaller but still positive effect (Fig. 3, see also Schwarz *et al*. 1998 and discussion of more specialized eusocial taxa below). Second, queen replacement might not always be entirely cost free, as assumed in our Primitively Eusocial model. While not causing complete group failure as Fu *et al*. assumed, queen death may sometimes lead to a temporary slow‐down in offspring production while workers compete to become the new queen. In some primitively eusocial taxa, a gerontocratic inheritance system where the new queen is always the oldest surviving female may largely avoid conflict during queen replacement (Bridge & Field 2007; see also Bang & Gadagkar 2012). However, Strassmann *et al*. (2004) found that some forms of aggression between nest‐mates increased and nest growth temporarily decreased following experimental removal of the queen from groups of paper wasps (see also Tsuji & Tsuji 2005). Natural queen deaths might not always have this effect if cues signifying queen ageing (Panek *et al*. 2001) enable replacement queens to prepare physiologically for their new roles in advance. Note that another possible cost of queen death, reduced reproductive potential (e.g. fertility) of replacement‐ vs. original queens, seems unlikely close to the origin of eusociality. Overall, empirical evidence indicates that routine queen replacement, and rates of offspring production that increase gradually with group size, are representative of primitively eusocial Hymenoptera.

Our analysis indicates that the risk‐return tradeoff identified by Fu *et al*. does not apply to the origin of eusociality. What, then, is the explanation for the apparent rarity with which eusociality has evolved (Fu *et al*. 2015)? The answer may be that no explanation is needed. What counts as rarity is debatable (Bourke 2011b; Liao *et al*. 2015), and eusociality cannot evolve in just any taxon. For example, ancestral provisioning of a nest is logically necessary before workers can boost the reproduction of a queen by provisioning her offspring, helping to explain the disproportionate number of origins of eusociality in the Hymenoptera. Nevertheless, eusociality has evolved more than 20 times in insects and an even greater number of times in vertebrates (Bourke 2011a; note that cooperative breeding is essentially equivalent to eusociality). A concrete comparison has been made with powered flight, which like eusociality is an ecologically very successful trait. Powered flight has evolved only four times, suggesting that conditions for the evolution of eusociality are at least 10 times less stringent (Bourke 2011b).

### The origin of more specialised castes

Eusocial taxa living in larger groups with morphologically specialized castes also do not appear to face a risk‐return tradeoff. There is again little evidence of a threshold group size below which workers are unproductive, and queen death does not generally lead to catastrophic group failure, although outcomes following queen death need further study (see Fig. S11). However, the birth rate may saturate (decline) in larger groups, reducing the probability that a eusocial genotype avoids extinction. Our results show that the phylogenetically widespread GSDQ strategy, where colony growth (worker production) precedes reproduction, has the potential to mitigate the effect of birth rate saturation if the switch to reproduction occurs at a group size matching the saturation point (Fig. 5). Empirical work is needed to determine how close the match actually is.

Our results also provide a new explanation for the evolution of GSDQ strategies generally. At the group level, GSDQ has previously been thought to optimize reproductive output when the growing season is finite: in seasonal environments where early offspring become workers while those produced later (in larger groups) enter hibernation and become queens the following year (Macevicz & Oster 1976; Mitesser *et al*. 2007). In such environments, it is also clear how GSDQ could be adaptive at the individual level – if offspring become workers earlier in the season (in smaller groups), they have more time left in which to be productive (Lucas & Field 2013). It is therefore notable that GSDQ improves the performance of our Primitively Eusocial strategy even in the completely aseasonal environment of our model. So long as workers in small groups are more productive than lone females (*b_∆_* > *b*_0_), earlier worker production (GSDQ's initially high staying ratio) makes it more likely that offspring take advantage of this greater productivity by becoming workers before the group fails. This helps to explain why some taxa that are restricted to less seasonal environments still exhibit GSDQ (e.g. tropical stingless bees, Meliponinae).

## Conclusion

Our analysis shows that assumptions about life history and ecology, not just genetic relatedness, matter critically when considering both the origin and elaboration of eusociality (see also Korb & Heinze 2008; Fromhage & Kokko 2011; Nonacs 2011, 2019; Avila & Fromhage 2015). The risk‐return tradeoff proposed at the origin of eusociality by Fu *et al*. (2015) is not robust to realistic assumptions. However, their general modelling framework is still potentially useful. For example, because it explicitly models lineage extinction, their approach could be used to compare eusociality with solitary nesting in terms of risk‐spreading. As a simple illustration, if we consider our Primitively Eusocial model with queen replacement, equal mortality rates for all individuals, and with *b_∆_* = *b*_0_ at all group sizes, we might expect eusociality and solitary nesting to perform equally well. Effectively, a eusocial group with *i* individuals is then no more than the sum of *i* solitary individuals. Indeed, we find that in this case the two genotypes have equal extinction probabilities. However, if we then add a low rate of group size‐independent whole nest failure, which might represent predators occasionally overwhelming the group, we find that the extinction probability for eusociality slightly exceeds that for solitary nesting. This is because the solitary strategy is effectively spreading risk in the sense of ‘not putting all your eggs in one basket’ (see section 7 of Supporting Information for more details). To see this, imagine a simple scenario where a nest‐initiating foundress always produces two offspring and then dies. If she has a eusocial genotype, the two offspring always stay in their natal nest and reproduce at rate 2*b*, whereas if she has the solitary genotype, each offspring initiates a separate nest and reproduces at rate *b*. Further imagine that before the offspring begin to reproduce, whole nest failure destroys a random half of the nests in the population. Half of the eusocial nests are destroyed and so have zero productivity, while the other half survive and have productivity 2*b*, giving a mean productivity of *b* for a eusocial foundress. For the solitary strategy, there is a 25% chance that both daughter nests fail (giving zero productivity), a 50% chance that one fails (giving *b*), and a 25% chance that neither fails (giving 2*b*). On average, productivity is again *b* for the solitary foundress, but the chance of zero productivity (extinction) is smaller under solitary nesting. Solitary foundresses effectively spread risk across more nests, lessening the risk of catastrophe before any offspring have been produced. Modelling extinction probabilities has the potential to be a generally useful tool to investigate risk‐spreading under different social life‐histories.

## Authorship

JF conceived the study and wrote the first draft of the manuscript. HT performed modelling work and wrote the first draft of the Supporting Information. Both authors interpreted output data and were fully involved in all aspects of the work.

## Data Availability

No new data were created or analysed in this study.
