# Supplementary material for: The evolution of eusociality: no risk‐return tradeoff but the ecology matters
Source: Ecol Lett. 2019 Dec 29;23(3):518–26. doi: 10.1111/ele.13452 (PMC7027560; doi:10.1111/ele.13452)
Supplement: Supplementary file 1 [file ELE-23-518-s001.pdf]

Supporting Information: A Markov Model of Eusociality at its Origin

Hiroshi Toyoizumi
Waseda University

Jeremy Field
University of Exeter

1. Introduction

We here extend the model of Fu *et al.* (2015) to include:

1. Worker mortality: we model not only queen but also workers' death rates.
2. Inheritance of nest: instead of queen death causing catastrophic group failure, we assume that if the queen dies a surviving worker can take over as the egg-layer so that the group continues to produce offspring.
3. Productivity related linearly to group size: rather than productivity increasing only above a threshold group size, we assume that each worker increases productivity by the same amount.

We use branching process (see Durrett (2012) for example) and transient Markovian arrival process (Latouche *et al.* 2003, He 2014) to analyze our models and to derive both the basic reproductive number and the extinction probability. We find that allowing inheritance is particularly important for understanding the origin of eusociality. Note that Toyoizumi & Field (2014) uses a similar technique using quasi-birth and death process but only to derive the basic reproductive number.

2. Solitary Model

We start with the basic solitary model, which introduces the concepts we will use. Like Fu *et al.* (2015), we assume that a solitary lineage starts with a single foundress that builds a nest and starts producing offspring. Birth intervals and the lifespan of the foundress are independent and exponentially distributed with b_0 being the offspring birth rate and d_0 being the death rate of the foundress. Here we assume $d_0 < b_0$. Letting X be the number offspring that the foundress produces in her life span, we have

$$P(X = n) = \frac{d_0}{b_0 + d_0} \left(\frac{b_0}{b_0 + d_0} \right)^n . \tag{1}$$

This can be interpreted as the following stochastically equivalent setting: set neutral random events in time with the rate $b_0 + d_0$, where each event is birth with the probability $b_0/(b_0 + d_0)$ or death with the probability $d_0/(b_0 + d_0)$, so that the number of births until foundress death follows a geometric distribution as in (1).

Using the expectation of X, we can find the basic reproductive number R as

$$R = E[X] = \sum_{n=0}^{\infty} n \frac{d_0}{b_0 + d_0} \left(\frac{b_0}{b_0 + d_0} \right)^n = \frac{b_0}{d_0},$$

which is simply the product of the average life span $1/d_0$ and the birth rate b_0. When R is greater than 1, one foundress produces more than one offspring, and the population grows exponentially.

In the solitary model, all offspring act as foundresses, start their own nests and begin reproducing immediately. The population is extinct when all foundresses are dead. We also investigate the population's extinction probability p, which can be analyzed by the probability generating function $\phi(z) = E[z^X]$ for $z \in [0, 1]$. The extinction probability p is known to be the minimal solution of the equation for the branching process (e.g. see Durrett (2012) for an example):

$$p = \phi(p) = E\left[p^X\right] . \tag{2}$$

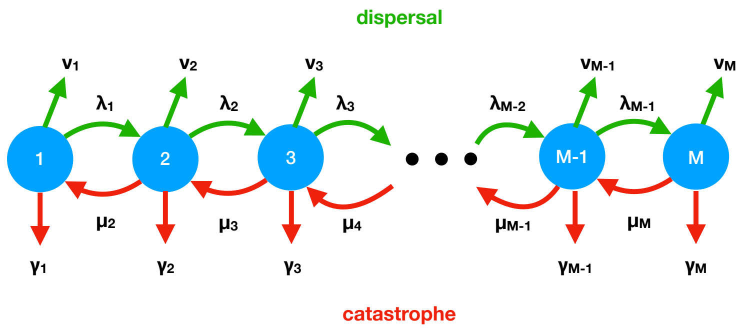

Figure S1: Transient Markov chain model of dispersal. Numbers in blue circles represent group size $L(t)$ (including the queen), which changes at the rates shown. Green arrows are related to the birth and red arrows are related to the death of individuals in the group or (downward-pointing red arrows) to catastrophic group failure. Note that upward-pointing green arrows indicating dispersal do not change the group size.

Equation (2) can be interpreted as follows: given that the initial nest produces X independent dispersing foundresses, all of these X lineages go extinct with probability p^X, and thus the extinction probability p is simply the expected probability $E[p^X]$. By (1), given $R = b_0/d_0 > 1$, we have

$$\phi(z) = E[z^X] = \sum_{k=0}^{\infty} z^k \frac{d_0}{b_0 + d_0} \left(\frac{b_0}{b_0 + d_0} \right)^k = \frac{d_0}{b_0 + d_0 - b_0 z}.$$

Thus, the extinction probability p has to satisfy

$$0 = p(b_0 + d_0 - b_0 p) - d_0 = (-b_0 p + d_0)(p - 1),$$

and it is easy to see that $p = d_0/b_0 = 1/R$ is its minimal solution. The extinction probability and the basic reproductive number are essentially the same in the solitary model, with a larger reproductive number R always corresponding to a smaller extinction probability p. However, we will see that there is not always such a simple relationship between extinction probability and the basic reproductive number in general eusocial models.

3. Markov Models of Eusociality and Examples

Consider an initial eusocial foundress who builds a nest and starts producing offspring. Under the eusocial strategy, some of her offspring may remain in the natal group as helpers, and we let $L(t)$ be the group size (i.e. the number of individuals in the group at time t including the foundress if she is still alive). We let X be the number of dispersing offspring (new foundresses) produced before the group termination time T that occurs either when no individual remains alive ($L(T) = 0$) or when a sudden catastrophic event happens to the group. We assume the group has a maximum size M.

For simplicity, we assume that the dynamics of $L(t)$ can be characterised by a Markov chain with four types of rate parameters $(\lambda_i, \mu_i, \gamma_i, \nu_i)$ which are determined specifically in each variant of our eusocial model as described in Section 4. This means that we focus on only the current group size $L(t)$ to predict the future dynamics, ignoring other factors such as larval development (see Figure S1). Suppose that group size $L(t) = i$. The group size increases with rate λ_i and decreases with rate μ_i. In addition, the nest terminates with rate γ_i due to some catastrophe (the queen's death in the model of Fu *et al.* (2015), or perhaps due to attack by predators in our bet-hedging model of Section 7). When $L(t) = 1$, a decrease in group size (death of the last remaining individual) always terminates the nest, so we set $\gamma_1 = \mu_1$. Also, at $L(t) = M$, all offspring are assumed to disperse,

so we set $\lambda_M = 0$. An offspring dispersal event occurs with rate ν_i, and each dispersed offspring is assumed to be a foundress that starts her own nest.

We use the expectation and the probability generating function of X to analyze the eusocial models as we did for the solitary model in Section 2. In order to introduce our method before specifying our focal models, we first specify some simplistic eusocial models, including one with no catastrophic events and another with no inheritance of the queen position. For a model incorporating both of these aspects simultaneously, we would need to use a transient Markov process, which is discussed in Section 6.

3.1. Eusocial model with no catastrophic events (except for group termination when the last individual dies)

In this model, no catastrophic events are allowed during periods when group size exceeds 1 ($\gamma_i = 0$ for $i \geq 2$), and the nest terminates when the last individual (queen, by definition) dies ($\mu_1 = \gamma_1$). Inheritance of the queen position is allowed. The basic reproductive number for this model $R_I = E[X]$ is estimated by,

$$R_I = \sum_{k=1}^{M} \left(\prod_{j=1}^{k-1} \frac{\lambda_j}{\mu_j} \right) \frac{\nu_k}{\mu_k}, \tag{3}$$

where we use the convention $\prod_{j=2}^{1} a_j = 1$. A brief intuitive explanation of (3) is as follows (see more rigorous treatment below). Each time group size $L(t)$ goes up to 2, it must eventually go back down to 1 because there are no catastrophic events that terminate groups larger than one individual. On average, upward visits to group size 2 are repeated λ_1/μ_1 times before the group terminates when the last individual dies, because of the lack of memory in the exponential total time spent at group size $L(t) = 1$. Similarly, after each upward visit to group size 2, $L(t)$ may increase to 3 and back down to 2 several times, but on average it repeats λ_2/μ_2 times. Thus, the expected total number of upward visits to size 3 is $(\lambda_1/\mu_1) \times (\lambda_2/\mu_2)$. Generally, the term $\prod_{j=1}^{k-1} \lambda_j/\mu_j$ represents the expected number of upward visits to group size k, while ν_k/μ_k is the expected number of dispersals at size k during each upward visit to k.

The probability generating function $\phi_I(z) = E[z^X]$ is calculated by

$$\phi_I(z) = \frac{\mu_1}{\mu_1 + \nu_1 + \lambda_1 - \nu_1 z - \lambda_1 \phi_2(z)}, \tag{4}$$

where $\phi_k(z)$ are obtained recursively in k starting from M as

$$\begin{aligned} \phi_k(z) &= \frac{\mu_k}{\mu_k + \nu_k + \lambda_k - \nu_k z - \lambda_k \phi_{k+1}(z)}, \\ \phi_M(z) &= \frac{\mu_M}{\mu_M + \nu_M - \nu_M z}. \end{aligned} \tag{5}$$

We give the derivation of (3) and(4) by breaking down the dispersals as follows. During periods when $L(t) = 1$, there are events such as dispersals (D), upward visits to group size 2 (U), and the termination of the group (T). Ignoring the type of event, events occur at rate $\nu_1 + \lambda_1 + \mu_1$, and the i-th event E_i turns out to be D with probability $\nu_1/(\nu_1 + \lambda_1 + \mu_1)$, for example. Let C_k be the time interval starting from k-th event of type U until the first subsequent return to group size 1, and let $N_1 = \sum_i 1_{(E_i=U)}$ be the number of these U events until the termination event T. Let Y_k be the number of dispersals during the interval C_k. Note that $Y_1, \ldots Y_{N_1}$ are stochastically identical and independent due to the assumption of the Markov property of $L(t)$. Let $X_1 = \sum_i 1_{(E_i=D)}$ be the number of dispersals during $L(t) = 1$, then:

$$X = X_1 + \sum_{k=1}^{N_1} Y_k. \tag{6}$$

The probability generating function of X should be estimated carefully because X_1 and N_1 are not independent, but the sum X_1+N_1 is a geometric random variable and $P(X_1+N_1=n)=\alpha_1^n(1-\alpha_1)$ where $\alpha_1=(\nu_1+\lambda_1)/(\nu_1+\lambda_1+\mu_1)$. Given that the total number of events X_1+N_1 equals l, each event is either D or U with the probability $\lambda_1/(\lambda_1+\nu_1)$ and $\nu_1/(\lambda_1+\nu_1)$ respectively. Because all events occur independently of each other, we have

$$\begin{aligned} E\left[z^{X_1}y^{N_1}\middle|X_1+N_1=l\right] &= \prod_{i=1}^{l} E\left[z^{1(E_i=D)}y^{1(E_i=U)}\middle|X_1+N_1=l\right] \\ &= \left\{\frac{\lambda_1}{\lambda_1+\nu_1}y+\frac{\nu_1}{\lambda_1+\nu_1}z\right\}^l. \end{aligned}$$

Unconditioning on X_1+N_1, we have the joint probability generating function of X_1 and N_1 as

$$\begin{aligned} E\left[z^{X_1}y^{N_1}\right] &= (1-\alpha_1)\sum_{l=0}^{\infty}\alpha_1^l\left\{\frac{\lambda_1}{\lambda_1+\nu_1}y+\frac{\nu_1}{\lambda_1+\nu_1}z\right\}^l \\ &= \frac{\mu_1}{\nu_1+\lambda_1+\mu_1-\nu_1 z-\lambda_1 y}. \end{aligned} \tag{7}$$

By (6) and the independence of Y_j with X_1 and N_1, we have

$$E\left[z^X\right]=E\left[E\left[z^X\middle|X_1,N_1\right]\right]=E\left[z^{X_1}\prod_{j=1}^{N_1}E\left[z^{Y_j}\middle|X_1,N_1\right]\right]=E\left[z^{X_1}\phi_2(z)^{N_1}\right],$$

where $\phi_2(z)=E[z^{Y_j}]$. Setting $y=\phi_2(z)$ in (7), we have (4). The decomposition of Y_j similar to (6) for larger nest sizes will recursively give (5). In addition, it is easy to see that (3) holds by checking $R_I=E[X]=d\phi_I(z)/dz|_{z=1}$.

3.2. Linear eusocial model

One particularly interesting case of the model in Section 3.1 is when birth and death rates increase linearly with group size, i.e. $b_k=kb_0$ where $\lambda_k+\nu_k=b_k$ and $d_k=\mu_k=kd_0$. In this case, eusociality is effectively just the aggregation of solitary individuals. Thus, measures of performance for eusociality should be the same as for solitary nesting. First considering the extinction probability, we find that this is the case. Starting from the maximum group size M ($\lambda_M=0$) in (5), we have

$$\phi_M(d_0/b_0)=\frac{Md_0}{Md_0+Mb_0-Mb_0/(d_0/b_0)}=d_0/b_0.$$

Recursively, if $\phi_{k+1}(d_0/b_0)=d_0/b_0$, again we have

$$\phi_k(d_0/b_0)=\frac{kd_0}{kd_0+kb_0-\nu_k/(d_0/b_0)-\lambda_k/(d_0/b_0)}=\frac{d_0}{d_0+b_0-b_0/(d_0/b_0)}=d_0/b_0.$$

Consequently, we can show that d_0/b_0 is the extinction probability for the linear eusocial model satisfying $d_0/b_0=\phi_I(d_0/b_0)$ with $d_0/b_0<1$. This is the same as for the solitary model in Section 2 (above).

However, now considering the basic reproductive number R, when $M=2$ in (3), we have

$$R_I=\frac{\nu_1}{\mu_1}+\frac{\lambda_1}{\mu_1}\frac{\nu_2}{\mu_2}=\frac{\nu_1}{d_0}+\frac{\lambda_1}{d_0}\frac{b_0}{d_0}\geq\frac{b_0}{d_0},$$

since $b_0/d_0>1$. This is just a consequence of additional dispersals being added into the basic reproductive number. In general, we can show that $R_I\geq b_0/d_0$ for $M\geq 3$. Thus, the basic reproductive number is always larger than for the solitary model, even though the two models are essentially equivalent. This indicates that the extinction probability is a preferable performance measure.

3.3. Eusocial model with no inheritance of the queen position and no downward transitions in group size

In our final illustrative model, we allow no inheritance or downward transitions in group size ($\mu_i = 0$ for $i \geq 2$), but we allow catastrophic events. The basic reproductive number, which is obtained as (16) in Fu *et al.* (2015) in slightly different form, is then equal to

$$R_F = \sum_{k=1}^{M} \left(\prod_{j=1}^{k-1} \frac{\lambda_j}{\gamma_j + \lambda_j} \right) \frac{\nu_k}{\gamma_k + \lambda_k},$$

where $\lambda_M = 0$. At any group size j, either the group next goes up to size $j+1$ or it is terminated. Thus, the term $\prod_{j=1}^{k-1} \lambda_j/(\gamma_j + \lambda_j)$ is the probability that the group reaches size k. The term $\nu_k/(\gamma_k + \lambda_k)$ is the number of dispersals while at size k.

The probability generating function can be obtained by

$$\phi_F(z) = E\left[z^X\right] = \sum_{k=1}^{M} \left(\prod_{j=1}^{k-1} \frac{\lambda_j}{\gamma_j + \lambda_j + \nu_j - z\nu_j} \right) \frac{\gamma_k}{\gamma_k + \lambda_k + \nu_k - z\nu_k}. \tag{8}$$

We give a brief derivation of (8) as follows. Consider the case where the group increases in size while producing X_i dispersing offspring at size i and finally terminates at size K. Then, by Markov property

$$P\left(X_1 = n_1, \ldots, X_k = n_k, K = k\right) = \prod_{j=1}^{k-1} \left(\frac{\nu_j}{\gamma_j + \lambda_j + \nu_j} \right)^{n_j} \frac{\lambda_j}{\gamma_j + \lambda_j + \nu_j} \left(\frac{\gamma_k}{\gamma_k + \lambda_k + \nu_k} \right)^{n_k} \frac{\gamma_k}{\gamma_k + \lambda_k + \nu_k}.$$

The total dispersals X can be decomposed by X_i, and we have

$$E\left[z^X\right] = \sum_{k=1}^{M} E\left[z^X 1_{\{K=k\}}\right] = \sum_{k=1}^{M} E\left[\prod_{j=1}^{k} z^{X_j} 1_{\{K=k\}}\right]$$

Then, we can obtain (8) by calculating the expectation.

4. Focal Eusocial Models

We now define the specific models shown schematically in Figure S2, including the Primitively Eusocial model we focus on in the main text. We solve all models numerically.

First, we define the model used in Fu *et al.* (2015), which has: (1) no worker mortality (2) no inheritance following queen death, and (3) a threshold-type offspring birth rate, and a threshold-type queen death rate which causes a catastrophic event. In contrast with the solitary model, newly-born offspring now remain in the natal group with probability q (the staying ratio), where they may help the queen to rear offspring. All newly-born offspring must leave the nest when $L(t) = M$. If group size reaches a threshold ($L(t) \geq m$), both the birth rate and the death rate change (positive effect of helpers). When the foundress dies, the group is terminated. Thus, in terms of our general model in Section 6, the Eusocial strategy of Fu et al. is described as:

$$\lambda_i = \begin{cases} qb_0 & \text{for } 1 \leq i < m, \\ qb & \text{for } m \leq i \leq M-1, \\ 0 & \text{for } i = M. \end{cases} \qquad \nu_i = \begin{cases} (1-q)b_0 & \text{for } 1 \leq i < m, \\ (1-q)b & \text{for } m \leq i \leq M-1, \\ b & \text{for } i = M. \end{cases} \tag{9}$$

$$\gamma_i = \begin{cases} d_0 & \text{for } 1 \leq i < m, \\ d & \text{for } m \leq i \leq M, \end{cases}$$

and $\mu_i = 0$ for all i (there is no mortality except catastrophic group failure following the death of the queen). Using this approach, we successfully reproduced the results shown in Fu *et al.* (2015) Figures 2 and 3.

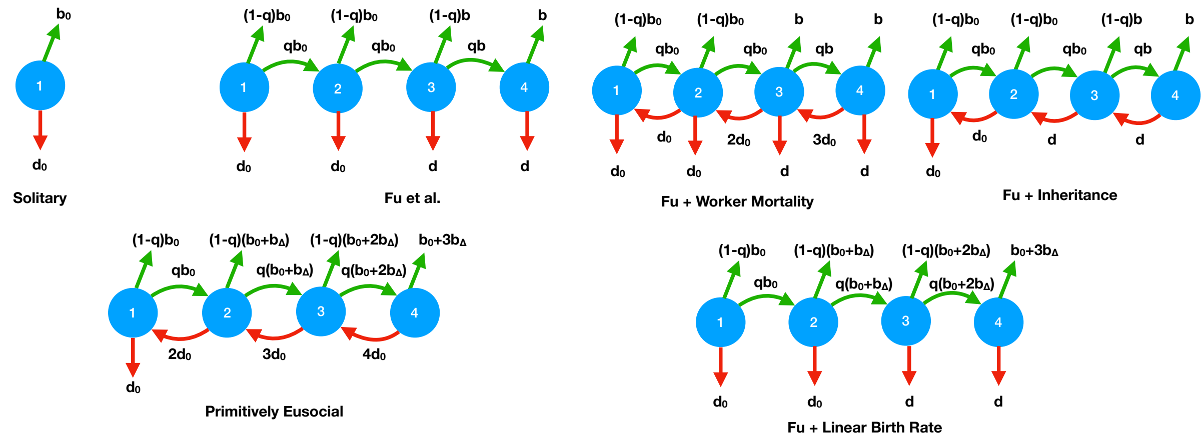

(a) Solitary, Fu et al. eusoicial and Prim Eusocial **(b)** Auxiliary Models

Figure S2: Models analysed (with $m = 3$ and $M = 4$).

We next specify our Primitively Eusocial model (Figure S2a). As in Fu et al.'s model, offspring stay in their natal groups with probability q, but in contrast with the threshold-type offspring birth rate in Fu et al.'s model, we incorporate a linear birth rate so that workers contribute something even at small group sizes. We also allow worker mortality at a rate proportional to group size, reflecting the cost of the linear increase in productivity. In contrast with Fu et al., when the foundress (queen) in the group dies, we allow inheritance of the egg-laying position: the group is not terminated and continues producing offspring (initially at the rate appropriate for the smaller group size) until the last individual in the group dies. Thus, our Primitively Eusocial strategy is described as:

$$\lambda_i = \begin{cases} q\{b_0 + (i-1)b_\Delta\} & \text{for } 1 \le i < M \\ 0 & \text{for } i = M. \end{cases} \qquad \nu_i = \begin{cases} (1-q)\{b_0 + (i-1)b_\Delta\} & \text{for } 1 \le i < M \\ b_0 + (M-1)b_\Delta & \text{for } i = M. \end{cases}$$

$$\gamma_i = \begin{cases} d_0 & \text{for } i = 1, \\ 0 & \text{for } 2 \le i \le M, \end{cases} \qquad \mu_i = \begin{cases} 0 & \text{for } i = 1, \\ d_0 i & \text{for } 2 \le i \le M, \end{cases}$$

where b_Δ represents the productivity boost provided by each additional worker.

To separately examine the effect of each of our modifications to Fu et al.'s Eusocial strategy model (worker mortality, inheritance, linear birth rate), we use three auxiliary models; (1) Fu + worker mortality, (2) Fu + inheritance, and (3) Fu + linear birth rate (see Figure S2b). Each represents a single component of our Primitively Eusocial model.

In the Fu + worker mortality model, with each additional worker we add d_0 to the downward group size transition rate. Thus, in (9), we add the downward transition rate as

$$\mu_i = (i-1)d_0,$$

where the factor -1 reflects the fact that foundress death results in whole group failure and counted in the catastrophe rate γ_i. Note that both μ_i and γ_i are positive so we need to use general model described in Section 6.

In the Fu + inheritance model, we modify Fu et al.'s Eusocial strategy by assuming that one of the remaining workers inherits the egg-laying position when the queen dies, so that there is no whole group catastrophe rate for $L(t) \ge 2$, and we set:

$$\gamma_i = \begin{cases} d_0 & \text{for } i = 1, \\ 0 & \text{for } i \ge 2, \end{cases} \qquad \mu_i = \begin{cases} d_0 & \text{for } 2 \le i < m, \\ d & \text{for } m \le i \le M, \end{cases}$$

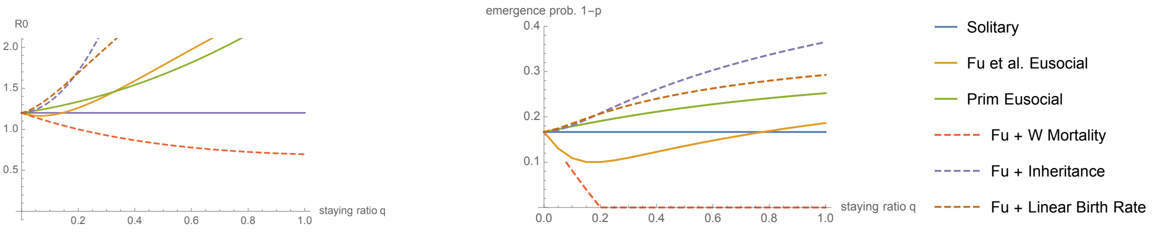

(a) Basic reproductive number R.   **(b)** Emergence probability $1-p$

Figure S3: Comparison of Models in terms of R and in terms of the probability of avoiding extinction (emergence probability). The parameters are set by $M = m = 3; b_0 = 0.12, d_0 = 0.1; b = 0.45, d = 0.05; b_\Delta = (b - b0)/(m-1) = 0.165$.

Note that this model is otherwise specified as in (4), i.e. we do not allow worker deaths.

Finally, in the Fu + linear birth rate model, we assume that group productivity increases linearly with the addition of each worker, so that:

$$\lambda_i = \begin{cases} q\{b_0 + (i-1)b_\Delta\} & \text{for } 1 \le i < M \\ 0 & \text{for } i = M. \end{cases} \qquad \nu_i = \begin{cases} (1-q)\{b_0 + (i-1)b_\Delta\} & \text{for } 1 \le i < M \\ b_0 + (M-1)b_\Delta & \text{for } i = M. \end{cases}$$

Otherwise, the model is specified as in (4). If we add all three modifications simultaneously, we obtain our full Primitively Eusocial model.

5. Numerical Examples

Numerical example derivations of the basic reproductive number R and the emergence probability $1-p$ (probability of avoiding extinction) are shown in Figure S3. The maximum group size is assumed to be small ($m = M = 3$), which seems more realistic, when modelling the origin of eusociality, than the assumption ($m = 3, M = 100$) in Fu *et al.* (2015). In our Primitively Eusocial model and the Fu + linear birth rate model, the increase in productivity with group size is assumed to be a linear interpolation between b_0 and b; thus we set $b_\Delta = (b - b0)/(m-1)$. Other parameters are identical to those in the middle graphs in Figure 2 of Fu *et al.* (2015).

In some of their numerical examples, Fu *et al.* (2015) found that both R and $1-p$ decreased at smaller staying ratios q (see the orange lines in Figure S3), which they interpreted as reflecting a risk inherent in eusociality: the opportunity cost of worker production delaying the production of offspring that immediately disperse. However, in our Primitively Eusocial model, both R and $1-p$ increase with q, so that this risk apparently disappears. We check what causes this difference by analysing the three auxiliary (partially modified) models; (1) Fu + worker mortality, (2) Fu + inheritance, and (3) Fu + linear birth rate (see Figures S3). Adding worker mortality is a big disadvantage for eusociality, while adding either inheritance or a linear birth rate are advantages (see Figure S3), and the sum of the latter two positive effects is larger than the negative effect of worker mortality, so that the 'risk' disappears in our overall Primitively Eusocial model.

For a detailed comparison of the models (Figure S4), we illustrate the number of dispersing offspring produced at each group size $L(t)$ when the staying ratio is small ($q = 0.1$) and large ($q = 0.7$). Also, we include the expected time duration at each size. First, consider the case when the staying ratio is small ($q = 0.1$). Because we allow worker mortality, group size reaches the maximum ($M = 3$) less frequently in the Primitively Eusocial model than in Fu et al.'s eusocial strategy, so that productivity at this group size is smaller (see also Fu + worker mortality). However, inheritance results in greater productivity at $L(t) = 1$ than in Fu et al.'s model (the nest always returns to group size 1 before group termination), and this is the main source of the improved performance of eusociality in the Primitively Eusocial model (and the Fu + inheritance model). Inheritance is important because it avoids the wasted investment in worker production that occurs when catastrophic group termination follows queen death in Fu et al.'s eusocial model. Instead

allowing productivity to increase linearly with group size does lead to increased dispersal at group size $L(t) = 2$, but it is a smaller effect (compare the total dispersals at $L(t) = 1$ and 2 of Fu + inheritance and Fu + linear birth rate).

Next, consider the case when the staying ratio is high ($q = 0.7$). Fu et al.'s eusocial strategy now spends longer at the maximum group size thanks to the low queen death rate. This advantage is significant because reaching the maximum ($M = 3$) is relatively easy now. At high staying ratios, Fu et al.'s eusocial strategy can therefore perform better in terms of the basic reproductive number $E[X]$ for some combinations of parameter values, as shown in Figure S3a.

To obtain an idea of the robustness of our results when maximum group size is larger, we also illustrate the number of dispersals when $m = 3, M = 10$ (see Figure S5). The results are qualitatively similar to those for $m = 3, M = 3$, but the emergence probability for the eusocial strategy of Fu et al. now continuously decreases with increasing q. The effect of allowing just inheritance is much larger than in the case of $m = 3, M = 3$, because more offspring can potentially become helpers when $M = 10$, so that more investment is wasted in Fu et al.'s eusocial strategy when catastrophic group failure occurs.

Further, we checked the robustness of our result by looking at different b/d ratios in Fig. S6, which clearly shows that the Primitively Eusocial strategy performs better than Fu et al.'s Eusocial strategy, especially at smaller staying ratios q. We also produced region graphs assuming m=3 but M=10 (not shown). For both R and the emergence probability, these graphs were similar to Fig. S6b, with Primitive Eusociality performing best of the three strategies above a critical b/d ratio and the solitary strategy performing best below the critical ratio.

6. Transient Markov Model for Estimating Dispersals

We can extend the above argument to more general models including group dynamics within a eusocial group. We use transient Markovian Arrival Process (MAP) (Latouche *et al.* 2003) for modeling the number of dispersals.

Let $D = (D(i,j))$ be the transition rate matrix of a general M-state Markov chain of $L(t)$, where the component $D(i,j)$ represents the transition rate from group size i to group size j. We decompose D by the transition with and without dispersal. Let D_0 be the transition rate matrix without dispersal, D_1 be the transition rate matrix with dispersal, and C be the transition rate matrix to catastrophe. To give an example, D_0 for Figure S1 is a tridiagonal matrix:

$$D_0 = \begin{pmatrix} -(\lambda_1+\gamma_1+\nu_1) & \lambda_1 & & & 0 \\ \mu_2 & -(\lambda_2+\gamma_2+\mu_2+\nu_2) & \lambda_2 & & \\ & \mu_3 & -(\lambda_3+\gamma_3+\mu_3+\nu_3) & \lambda_3 & \\ & & & \ddots & \\ 0 & & & \mu_M & -(\gamma_M+\mu_M+\nu_M) \end{pmatrix}$$

and D_1 is a diagonal matrix with $D_1 = diag(\nu_1, \nu_2, \dots, \nu_M)$ and C is also a diagonal matrix with $C = diag(\gamma_1, \gamma_2, \dots, \gamma_M)$.

Since there can be a catastrophic event, $L(t)$ is transient, and both $D = D_0 + D_1$ and D_0 are non-singular, where we assume that a catastrophe and a dispersal do not occur at the same time. Note that we could potentially apply this analysis to more general models than Figure S1, such as when two or more births can occur at the same time.

The transient probability distribution $\pi_i(t) = P(L(t) = i, \text{no catastrophe during } (0,t))$ is characterized by the differential equation $\frac{d}{dt}\pi(t) = \pi(t)D$ and its solution:

$$\pi(t) = \pi(0)e^{Dt},$$

where $\pi(t) = (\pi_1(t), \dots, \pi_M(t))$ and $e^{Dt} = \sum_{k=0}^{\infty}(Dt)^n/n!$. In general, e^{Dt} is called the semi-group operator of the system that can advance the clock of the model. Since the Markov chain generated by D stops (terminates) at the catastrophic event, $\sum_{i=1}^{M}\pi(t)$ may not attain 1. The

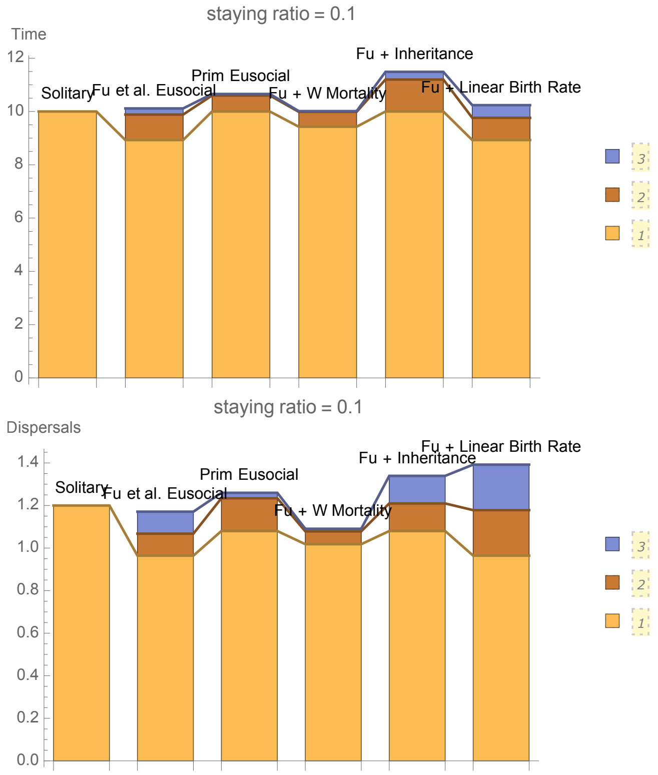

(a) $q = 0.1$

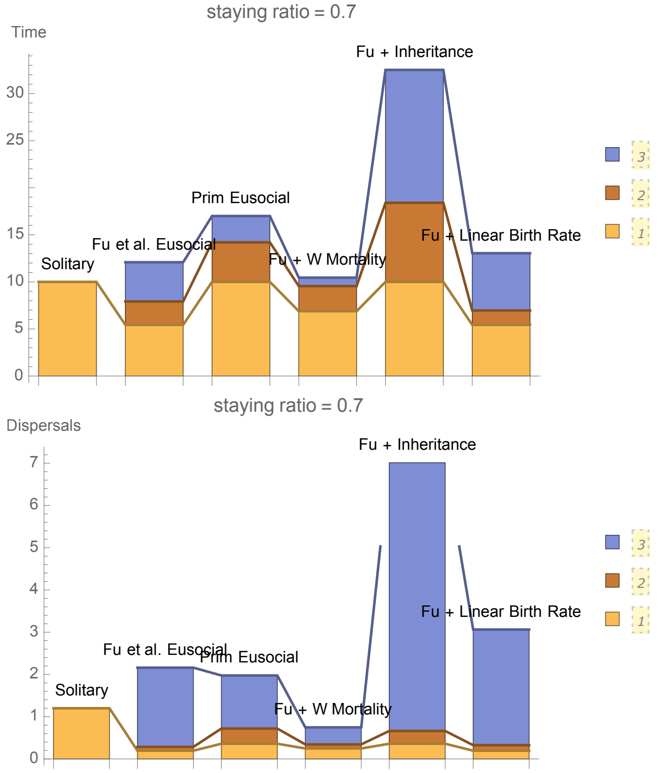

(b) $q = 0.7$

Figure S4: Mean number of dispersals and mean time spent at each group size, where colours represent different group sizes ($q = 0.1$ or $0.7; M = m = 3; b_0 = 0.12, d_0 = 0.1; b = 0.45, d = 0.05; b_\Delta = (b - b0)/(m - 1) = 0.165$)

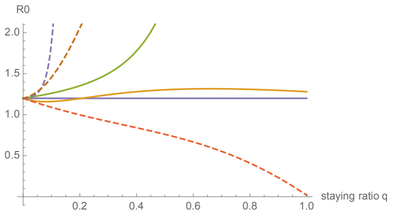

(a) Basic reproductive number R.

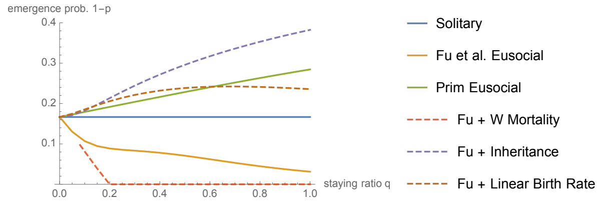

(b) Emergence probability $1 - p$

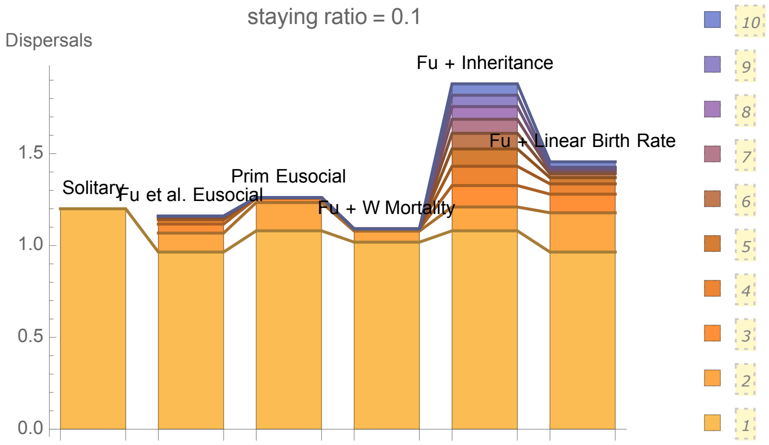

(c) Mean dispersals by group size

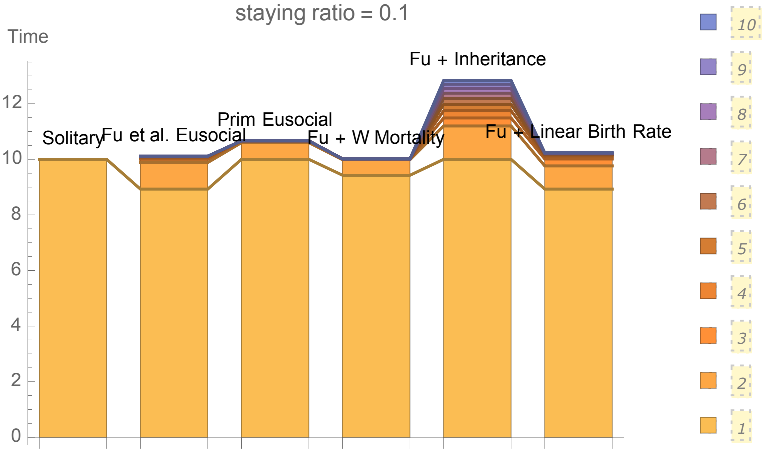

(d) Mean time spent in each group size

Figure S5: Comparison of Models as in Fig. S3 and S4a, but with $M = 10$. The parameters are set by $m = 3, M = 10; b_0 = 0.12, d_0 = 0.1; b = 0.45, d = 0.05; b_\Delta = (b - b0)/(m - 1) = 0.165$.

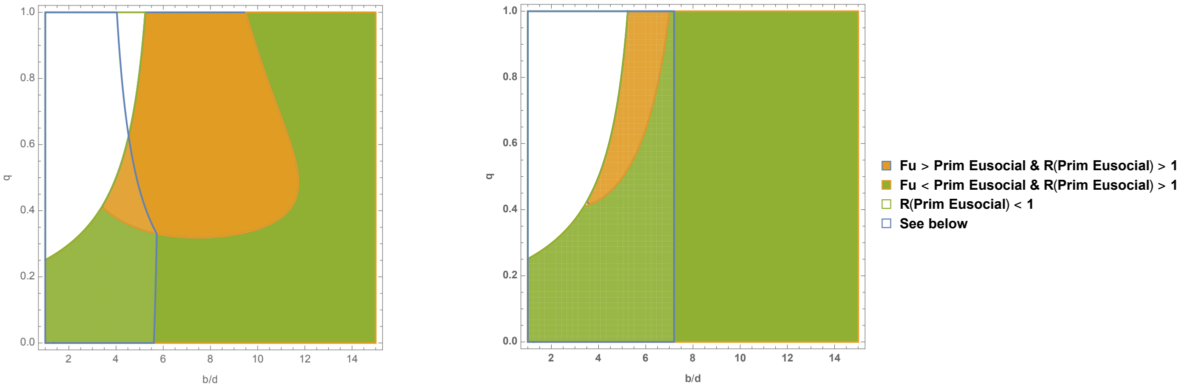

(a) Basic reproductive number R.

(b) Emergence probability $1-p$

Figure S6: Region graphs comparing Fu et al.'s solitary and eusocial strategies with our Primitively Eusocial strategy in terms of basic reproductive number and emergence probability in the $b/d-q$ plane. Other parameter values are $m=3, M=3; b_0=0.12, d_0=0.1; d=0.05; b_\Delta=(b-b0)/(m-1)$. The white region indicates the area where the Primitively Eusocial strategy is certain to go extinct (its basic reproductive number is $R=E[X]<1$). The area surrounded by the blue boundary is where the solitary strategy performs best so that in (b), for example, the solitary strategy performs best at all q values when b/d is low, whereas Primitive Eusociality performs the best when b/d is high.

missing quantity $1-\sum_{i=1}^{M}\pi(t)$ represents the probability that the nest has already terminated by the time t.

Let T_i be the time duration that the group size is i, then its expectation can be obtained by

$$E[T_i]=E\left[\int_0^\infty 1_{\{L(t)=i\}}dt\right]=\int_0^\infty \pi_i(t)dt=(\pi(0)(-D)^{-1})_i,$$

which is equivalent to the lifespan $1/d_0$ in the solitary model.

Since the Markov chain generated by D_0 represents the process stopped either at the catastrophe or dispersal, similar arguments give its semi-group operator as

$$P(L(t)=j,\text{no dispersal and no catastrophe during }(0,t)|L(0)=i)=(e^{D_0t})_{i,j}.$$

Let $F=\int_0^\infty e^{D_0t}D_1dt=(-D_0)^{-1}D_1$, which can be interpreted as the transition probability matrix on the successive events of dispersal. Note that F is a sub-stochastic matrix (at least one of the sums of each row is less than 1) since there is the potential for a catastrophic event. Similarly, we define $G=(-D_0)^{-1}C$ which is the transition probability matrix from dispersal to catastrophe. The matrices F and G corresponds to $b_0/(b_0+d_0)$ and $d_0/(b_0+d_0)$ in the solitary model (see Figure S7). Then,

$$(P(X=n,L(T)=1),\ldots,P(X=n,L(T)=M))=\pi(0)F^nG. \tag{10}$$

where $\pi(0)=(1,0,\ldots,0)$. The right-hand side of (10) can be regarded as starting from the initial distribution $\pi(0)$, repeating the dispersal transition (F) n times and then a catastrophe transition G occurs. It is not difficult to see that

$$P(X=n)=\pi(0)F^nG\mathbf{1}, \tag{11}$$

where $\mathbf{1}=(1,1,\ldots,1)^T$. This is indeed equivalent to Theorem 4.1 in Latouche *et al.* (2003) with $f_0=G\mathbf{1}$ and $f_1=0$ for the case of no catastrophe occurring at the same time as another event. Since F is sub-stochastic matrix, we have the identity $(I-zF)\sum_{n=0}^{\infty}z^nF^n=I$. Thus, we have

$$E\left[z^X\right]=\sum_{n=0}^{\infty}z^nP(X=n)=\pi(0)(I-zF)^{-1}G\mathbf{1}.$$

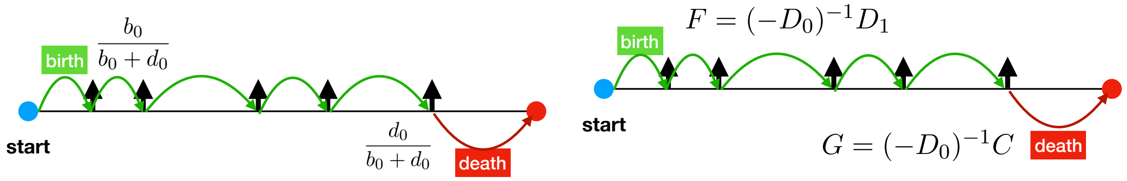

Figure S7: The dynamics of the solitary model (left) and the transient Markov model (right), illustrating how the matrix calculation is a direct extension of the solitary model. Starting from the left, each nest produces 5 dispersing individuals (black arrows) one at a time (dispersal events at the same nest are connected by green arrows), and then is terminated (red arrows). In the solitary model, $P(X=5) = \frac{d_0}{b_0+d_0}\left(\frac{b_0}{b_0+d_0}\right)^5$ as in (1). In the transient Markov model, by keeping track of the group size $L(t)$ using matrix calculations, $P(X=5) = \pi(0)F^5G\mathbf{1}$ as in (11). The two models coincide when $M=1$.

Also, since $(I-F)\sum_{n=0}^{\infty} nF^n = \sum_{n=1}^{\infty} F^n$, the basic reproductive number is obtained by

$$R = E[X] = \pi(0)F(I-F)^{-2}G\mathbf{1}.$$

Note that these expressions are the direct extension of the solitary case. Using these expressions, we can easily derive the basic reproductive number R and the extinction probability p in general models such as the one shown in Figure S1, especially for a small M.

7. Bet-Hedging

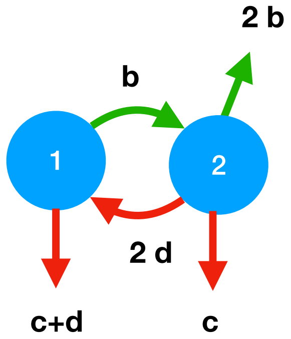

Figure S8: Schematic for a simple eusocial model to illustrate one form of bet-hedging

We also view our general model in terms of spatial bet-hedging (Starrfelt & Kokko 2012), where the solitary strategy effectively spreads risk by producing offspring that disperse individually as opposed to staying on the natal nest under the eusocial strategy. To illustrate this, we consider a simple case of the Primitively Eusocial model with $M=2$ and $q=1$ (all offspring become helpers below the maximum group size). We additionally specify completely linear productivity $b_\Delta = b_0$ and a rate of catastrophic group failure c that is independent of group size (see Figure S8):

$$D_0 = \begin{pmatrix} -(d+b+c) & b \\ 2d & -(2d+2b+c) \end{pmatrix}, D_1 = \begin{pmatrix} 0 & 0 \\ 0 & 2b \end{pmatrix}, C = \begin{pmatrix} c+d & 0 \\ 0 & c \end{pmatrix}.$$

Then, with some direct matrix calculations as in Section 6, the probability generating function for the number of dispersals X in this model is obtained by

$$E[z^X] = \frac{bc + (c+d)(c+2d) + 2b(c+d)(1-z)}{bc + (c+d)(c+2d) + 2b(c+d+b)(1-z)}, \quad (12)$$

and also the basic reproductive number is obtained by

$$R_{\text{eusocial}} = E[X] = \frac{2b^2}{bc + (c+d)(c+2d)}. \tag{13}$$

We compare these results with the solitary model under the same catastrophe rate c.

Before comparing the two strategies, however, we check the results in the case with a zero catastrophe rate: these should be the same for solitary and eusocial. As described in Section 2, given $b > d$, we have the extinction probability $p = d/b$ and the basic reproductive number $E[X] = b/d$ for the solitary model. If we set $c = 0$ in (12), we have

$$E[z^X] = \frac{2d^2 + 2bd(1-z)}{2d^2 + 2b(b+d)(1-z)},$$

and set $z = d/b$ to have

$$E[z^X]|_{z=d/b} = \frac{2bd^2 + 2bd(b-d)}{2bd^2 + 2b(b+d)(b-d)} = \frac{d}{b}.$$

This shows that the eusocial model has the same extinction probability as solitary model as predicted. However, if we set $c = 0$ in (13), we have

$$R_{\text{eusocial}} = \left(\frac{b}{d}\right)^2 > \frac{b}{d} = R_{\text{solitary}},$$

thus the eusocial model has always a larger basic reproductive number. This is because the lifespans of the two types of group are different, and suggests that generally, comparing the strategies in terms of the basic reproductive number could be misleading. Using the extinction probability is preferable.

Next, we compare the two strategies including a positive catastrophe rate. Given $b > c + d$, we have the extinction probability $p = (d + c)/b$ and the basic reproductive number $E[X] = b/(c + d)$ for the solitary model with catastrophe. If $b > c + d$, the simple eusocial model has

$$R_{\text{eusocial}} = \frac{2b^2}{bc + (c+d)(c+2d)} > \frac{2b^2}{bc + b(c+2d)} = \frac{b}{c+d} = R_{\text{solitary}},$$

thus the eusocial model has always a larger basic reproductive number, but this may not be meaningful as just discussed. For the extinction probability, taking $z = (d + c)/b$ in (12), then we have

$$\begin{aligned} E[z^X]|_{z=(d+c)/b} &= \frac{bc + (c+d)(c+2d) + 2(c+d)(b-c-d)}{bc + (c+d)(c+2d) + 2(b+c+d)(b-c-d)} \\ &= \frac{bc + (c+d)(2b-c)}{c(b-c-d) + 2b^2}. \end{aligned}$$

Now given $b > d + c$, we have

$$E[z^X]|_{z=(d+c)/b} - \frac{d+c}{b} = \frac{c(b-c-d)^2}{b\left\{2b^2 + c(b-c-d)\right\}} > 0.$$

Since $E[z^X]$ is a decreasing function of z, the extinction probability satisfying $p = E[p^X]$ is greater than $(d + c)/p$. Thus, the extinction probability for the simple eusocial model is always larger than for the solitary model. The solitary strategy performs better if we have a positive catastrophe rate, and the only difference between the two strategies is the fact that offspring produced under the solitary strategy initiate new nests.

Figure S9 shows the basic reproductive number and the extinction probability for the case of $M = 3$. We can see that as shown above for the case when $M = 2$, the solitary strategy performs slightly better than eusociality in terms of the emergence probability. However, the advantage is marginal, and so may be overcome by only a slightly super-linear productivity for the eusocial strategy.

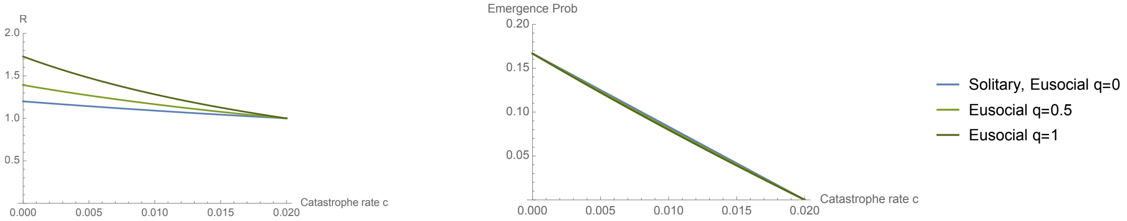

(a) Basic reproductive number R. **(b)** Emergence probability $1 - p$

Figure S9: An example where the solitary strategy has an advantage through risk-spreading. The parameters are set by $M = 3; b = 0.12, d = 0.1$.

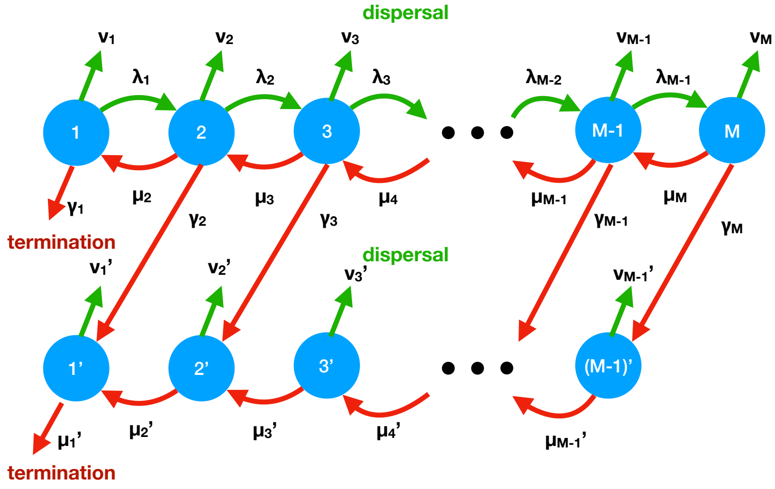

Figure S10: Transient Markov chain model with additional states following queen death (compare with Figure S1). The upper row of blue circles represents group sizes with the queen present, and the lower row of blue circles represents group sizes without the queen.

8. Model with No Queen Replacement and Partial Truncation of Worker Productivity Following Queen Death

We here extend the Markov model in Section 3 to include the following features of some advanced eusocial taxa: (1) no queen replacement is allowed; (2) following queen death, remaining workers produce offspring at a lower rate than before, and all of these offspring disperse so that group size decreases until the group terminates; and (3) queen lifespan exceeds worker lifespan. In this model, states after queen death are denoted by $1', 2', \ldots M-1'$ representing the number of workers remaining (see Figure S10). In state i', all new offspring are assumed to disperse with the rate ν_i', and the workers die with the rate μ_i'.

Let X be the number of dispersals, including dispersals from groups without a queen. We modify (4) to obtain the probability generating function of X as

$$E[z^X] = \psi_1(z) = \frac{\gamma_1 + \lambda_1 \psi_2(z)}{\gamma_1 + \nu_1 + \lambda_1 - \nu_1 z - \lambda_1 \phi_2(z)},$$

where $\phi_k(z)$ and $\psi_k(z)$ are obtained recursively in k starting from M as

$$\begin{aligned}
\phi_k(z) &= \frac{\mu_k}{\mu_k + \nu_k + \lambda_k + \gamma_k - \nu_k z - \lambda_k \phi_{k+1}(z)}, \\
\psi_k(z) &= \frac{\gamma_k \psi'_{k-1}(z) + \lambda_k \psi_{k+1}(z)}{\mu_k + \nu_k + \lambda_k + \gamma_k - \nu_k z - \lambda_k \phi_{k+1}(z)}, \\
\psi'_k(z) &= \prod_{i=1}^{k} \frac{\mu'_k}{\mu'_k + \nu'_k + \nu'_k z}, \\
\phi_M(z) &= \frac{\mu_M}{\mu_M + \nu_M + \gamma_M - \nu_M z}, \\
\psi_M(z) &= \frac{\gamma_M \psi'_{M-1}(z)}{\mu_M + \nu_M + \gamma_M - \nu_M z}.
\end{aligned} \tag{14}$$

We give a brief derivation of these equations using the concept of the fundamental period in quasi-birth and death processes (Neuts 1994, He 2014). The idea of the fundamental period is to decompose the process while the queen is alive into hierarchical periods, each of which starts from the time when the group reaches size k and ends at the first time instance when the group size falls below k (reaches $k-1$ and lower). During one such fundamental period at group size k, we may have several sub-fundamental periods at group size $k+1$.

At group size k, there may be some births, which may produce dispersing offspring, but if a new offspring stays in the group, then the group size reaches $k+1$. In a fundamental period at group size k, let X_k be the number of dispersals, and let N_k be the number of upward transitions to group size $k+1$. In our model, these fundamental periods can be classified into three categories according to how they end: (1) $E_{k,w}$ that end with the death of workers at group size k, (2) $E_{k,q}$ that end with death of the queen at group size k, and (3) $E_{k+,q}$ that end with the death of queen at any group size larger than k. Using similar arguments to those used to derive (7), we have the joint probability generating function of X_k and N_k as

$$\begin{aligned}
E\left[z^{X_k} y^{N_k} 1_{E_{k,w}}\right] &= \frac{\mu_k}{\nu_k + \lambda_k + \mu_k + \gamma_k - \nu_k z - \lambda_k y}, \\
E\left[z^{X_k} y^{N_k} 1_{E_{k,q}}\right] &= \frac{\gamma_k}{\nu_k + \lambda_k + \mu_k + \gamma_k - \nu_k z - \lambda_k y}, \\
E\left[z^{X_k} y^{N_k} 1_{E_{k+,q}}\right] &= \frac{\lambda_k \{P(E_{k+1,q}) + P(E_{(k+1)+,q})\}}{\nu_k + \lambda_k + \mu_k + \gamma_k - \nu_k z - \lambda_k y}.
\end{aligned}$$

First, decompose X using the fundamental periods for group size 2. Let X_1 be the number of dispersals at size 1, and let $Y_{2,n}$ be the number of dispersals during the n-th fundamental period at group size 2. Let Z be the number of births after queen death, all of which lead to offspring dispersal. Then, we have

$$X = X_1 + \sum_{n=1}^{N_1} Y_{2,n} + Z,$$

where N_1 is the total number of the fundamental periods at group size 2. Since Z but not $Y_{2,n}$ is

affected by how the last fundamental period ends, we have

$$
\begin{aligned}
E\left[z^X\right] &= E\left[z^X 1_{E_{1,q}}\right] + E\left[z^X 1_{E_{1+,q}}\right] \\
&= E\left[E\left[z^X 1_{E_{1,q}} \middle| X_1, N_1\right]\right] + E\left[E\left[z^X 1_{E_{1+,q}} \middle| X_1, N_1\right]\right] \\
&= E\left[z^{X_1} \prod_{j=1}^{N_1} E\left[z^{Y_{2,j}} 1_{E_{2,w}}\right]\right] + E\left[z^{X_1} \prod_{j=1}^{N_1} E\left[z^{Y_{2,j}} 1_{E_{2,w}}\right] z^{Y_2+Z} 1_{E_{1+,Q}}\right] \\
&= E\left[z^{X_1} \phi_2(z)^{N_1}\right] + E\left[z^{X_1} \phi_2(z)^{N_1}\right] \lambda \psi_2(z) \\
&= \frac{\gamma_1 + \lambda_1 \psi_2(z)}{\gamma_1 + \nu_1 + \lambda_1 - \nu_1 z - \lambda_1 \phi_2(z)},
\end{aligned}
$$

where

$$
\begin{aligned}
\phi_2(z) &= E\left[z^{Y_2} 1_{E_{2,w}}\right] \\
&= \frac{\mu_2}{\mu_2 + \nu_2 + \lambda_2 + \gamma_2 - \nu_2 z - \lambda_2 \phi_3(z)},
\end{aligned}
$$

and

$$
\begin{aligned}
\psi_2(z) &= E\left[z^{Y_2+Z}\{1_{E_{2,q}} + 1_{E_{2+,q}}\}\right] \\
&= \frac{\gamma_2 \psi_1'(z) + \lambda_2 \psi_3(z)}{\mu_2 + \nu_2 + \lambda_2 + \gamma_2 - \nu_2 z - \lambda_2 \phi_3(z)}, \\
\psi_1'(z) &= E[z^Z 1_{E_{2,q}}] = \frac{\mu_1'}{\mu_1' + \nu_1' + \nu_1' z}.
\end{aligned}
$$

Similar arguments hold for group sizes larger than 1, to produce (14).

Using this method, we extend the eusocial models in Section 4 to the following model incorporating features of advanced eusocial groups (with staying ratio q):

$$
\lambda_i = \begin{cases} q\{b_0 + (i-1)b_\Delta\} & \text{for } 1 \le i < M \\ 0 & \text{for } i = M, \end{cases} \qquad \nu_i = \begin{cases} (1-q)\{b_0 + (i-1)b_\Delta\} & \text{for } 1 \le i < M \\ b_0 + (M-1)b_\Delta & \text{for } i = M, \end{cases}
$$

$$
\gamma_i = \gamma, \qquad \mu_i = \begin{cases} 0 & \text{for } i = 1 \\ d_0(i-1) & \text{for } 2 \le i \le M, \end{cases}
$$

$$
\begin{aligned}
\nu_i' &= \{b_0 + (i-1)b_\Delta\}/2, \\
\mu_i' &= d_0 i,
\end{aligned}
$$

where $\gamma < d_0$ represents the lower death rate of the queen, and where we assume that the productivity of the group without the queen is reduced by half, but the worker death rate remains the same as when the queen is still present.

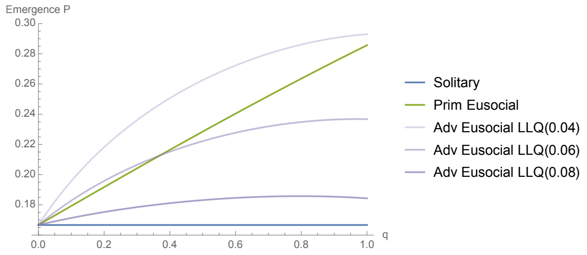

Figure S11: Primitively Eusocial and Solitary strategies compared with the corresponding advanced eusocial model specified in Figure S10 with three queen death rates ($\gamma = 0.04$ (upper purple line), 0.06 (middle), 0.06 (lower line)) and with worker productivity halved (to 0.06) following queen death. Other parameters are $M = 100; b_0 = 0.12, d_0 = 0.1; b_\Delta = 0.165$.

Figure S11 shows the emergence probability under this model for a relatively large maximum group size of $M = 100$. We can see that depending on the queen's death rate γ, the emergence probability may exceed or be less than the emergence probability for the Primitively Eusocial strategy described earlier in Section 4.

References

Durrett, R. (2012). *Essentials of stochastic processes.* Springer Science & Business Media.

Fu, F., Kocher, S.D. & Nowak, M.A. (2015). The risk-return trade-off between solitary and eusocial reproduction. *Ecol. Lett.*, 18, 74–84.

He, Q.M. (2014). From the poisson process to markovian arrival processes. *Fundamentals of Matrix-Analytic Methods*, pp. 85–154.

Latouche, G., Remiche, M.A., Taylor, P. *et al.* (2003). Transient markov arrival processes. *Ann. Appl. Probab*, 13, 628–640.

Neuts, M.F. (1994). *Matrix-geometric solutions in stochastic models: an algorithmic approach.* Dover Publishing.

Starrfelt, J. & Kokko, H. (2012). Bet-hedging—a triple trade-off between means, variances and correlations. *Biol. Rev*, 87, 742–755.

Toyoizumi, H. & Field, J. (2014). Dynamics of social queues. *J. Theor. Biol.*, 346, 16 – 22.
